# Rebuilding oral health systems in post-conflict Syria: a call for integration, innovation, and sustainability

**DOI:** 10.3389/fdmed.2026.1913447

**Published:** 2026-08-10

**Authors:** Humam Dawood, Nabil Kochaji, Julian Fisher

**Affiliations:** 1 Doctors of The World/ Médecins du Monde Germany, Munich, Germany; 2 Institute for Ethics and History of Health in Society, University of Augsburg, Augsburg, Germany; 3 Department of Oral Pathology, Faculty of Dental Medicine, Damascus University, Damascus, Syria; 4 Center for Integrative Global Oral Health, University of Pennsylvania l School of Dental Medicine, Philadelphia, PA, United States

**Keywords:** oral health system, post-conflict, sustainable healthcare, Syria, system rebuild

## Abstract

Oral health is a vital yet often overlooked component of health system recovery in conflict and post-conflict settings, despite its importance for population well-being, resilience, and social stability. In Syria, more than a decade of conflict has severely disrupted health infrastructure, displaced oral health professionals, and weakened service delivery, exacerbating the burden of untreated oral diseases amid increasing environmental and climate-related stressors. This Perspective explores how Syria could rebuild its oral health system within planetary boundaries, positioning oral health as an integral element of climate-resilient healthcare systems, human security, and health emergency preparedness and response. Drawing on the WHO Global Oral Health Action Plan (2023–2030), the UN 2030 Agenda for Sustainable Development, and lessons from Syria's crisis response, the paper proposes system-level reforms that integrate sustainability, climate resilience, and preparedness across governance, service delivery, workforce education, and supply chains. Key strategic priorities include embedding oral health within primary care and school health programs, promoting prevention-oriented and community-based approaches, adopting climate-resilient and environmentally responsible infrastructure, and institutionalizing emergency preparedness and transdisciplinary competencies within dental education and professional training. Rebuilding oral health systems in post-conflict Syria through a planetary health and human security lens offers a pathway to strengthen health system resilience, safeguard essential services under crisis conditions, and contribute to broader health, environmental, and socioeconomic recovery objectives, with relevance for other fragile and climate-vulnerable settings.

## 1 Introduction

Oral health is an essential yet often neglected component of health systems, particularly in fragile and conflict-affected settings. The thirteen years long conflict in Syria has devastated health infrastructure, displaced a significant portion of the dental workforce, and led to a surge in oral diseases (1, 2).

Globally, nearly one in four people live in fragile, conflict-affected, and vulnerable (FCV) settings, where weak health systems and disrupted services contribute to disproportionately high burdens of disease and mortality (3). These settings account for over 70% of epidemic-prone diseases, 60% of preventable maternal deaths, and nearly half of all infant and child deaths under five (3). Access to essential health services, including oral health, is a critical factor for people to rebuild their lives and livelihoods post-conflict.

The World Health Organization (WHO) has called for a strengthened global architecture for health emergency prevention, preparedness, response, and resilience (HEPR), and urges countries to adopt integrated, system-wide approaches that include all essential services (4). Human security is a people-centered approach focusing on protecting individuals from a wide range of threats—such as poverty, conflict, and disease—rather than just protecting state borders. It emphasizes freedom from fear, freedom from want, and freedom from indignity, aiming to ensure safety, dignity, and sustainable development (5).

Oral health, often siloed from broader health planning and processes, must be positioned as a core element of this agenda. In Syria, this means embedding oral health into national recovery and emergency preparedness strategies and programmes that are aligned with the WHO Global Oral Health Action Plan (2023–2030) (6).

This Perspective advances a normative argument grounded in WHO policy frameworks, published Syria-specific and fragile-setting literature, and the authors' professional experience within the Syrian dental sector; where published evidence is limited.

## 2 Conceptual framework: oral health within planetary boundaries, climate resilience, and human security

**Figure 1:**
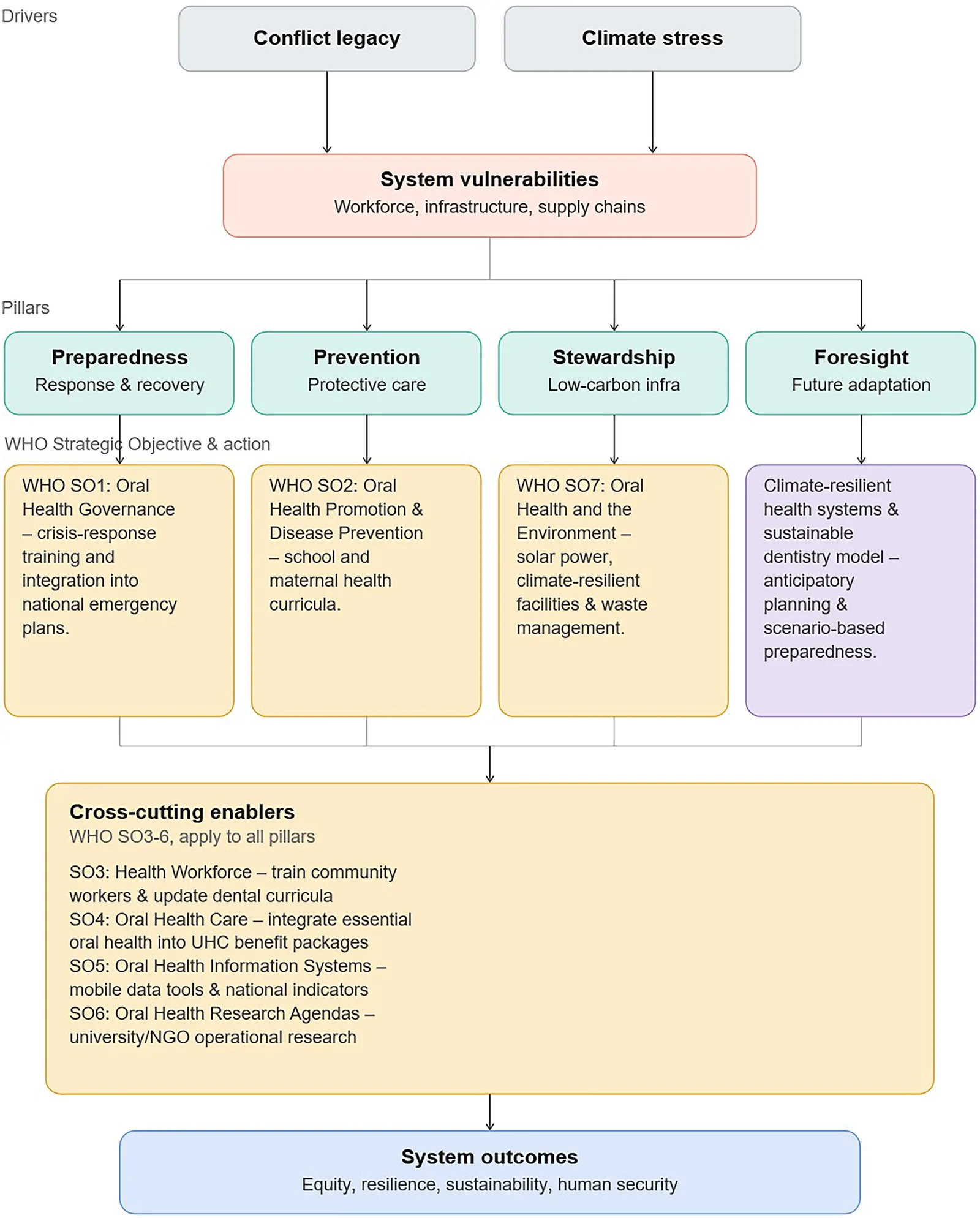
Conceptual framework for sustainable, resilient oral health system recovery in post-conflict Syria, aligned with the WHO global oral health action plan (2023–2030) and the Bangkok declaration—No health without oral health (2024). Conflict and climate stress drive vulnerabilities in workforce, infrastructure, and supply chains. Four pillars address these: preparedness (SO1), prevention (SO2), and stewardship (SO7) map to WHO Strategic Objectives; foresight draws instead on climate-resilient health systems and sustainable dentistry frameworks. SO3–6 (workforce, care, information systems, research) are cross-cutting enablers. All converge on equity, resilience, sustainability, and human security.

Oral health systems do not operate independently of Earth's systems that sustain human health. The framework of oral and planetary health emphasizes that human well-being is inseparable from the stability of Earth's natural systems, including climate regulation, freshwater availability, biodiversity, and sustainable resource use. In this model, sustainability operates as an overarching principle that integrates preventive, community-based care; responsible use of natural resources and environmentally sound waste management; preparedness for and recovery from health emergencies, natural disasters, and crises; and the adoption of anticipatory, future-oriented practices that support climate adaptation and mitigation. These elements constitute four pillars of a sustainable, resilient oral health system framework proposed in this Perspective: preparedness, prevention, stewardship, and foresight. Three pillars are anchored to specific WHO Strategic Objectives affirmed in the Bangkok Declaration—No Health Without Oral Health (2024), which extended the original six objectives of the Global Oral Health Action Plan (2023–2030) with a seventh dedicated to environmental sustainability (7). Preparedness aligns with Strategic Objective 1 (Oral Health Governance), operationalized through crisis-response training and integration of oral health into national emergency plans; prevention aligns with Strategic Objective 2 (Oral Health Promotion and Disease Prevention), through school- and maternal-based health programs; and stewardship aligns with Strategic Objective 7 (Oral Health and the Environment), through solar power adoption, climate-resilient facility design, and sustainable waste management. The fourth pillar, foresight, is not drawn from a WHO Strategic Objective but from the WHO operational framework for climate-resilient health systems and the model of sustainable dentistry, reflecting anticipatory, scenario-based planning for climate mitigation and adaptation. Strategic Objectives 3 through 6—health workforce, oral health care, oral health information systems, and oral health research agendas—function as cross-cutting enablers supporting all four pillars rather than any single one. Figure 1 illustrates how these pillars and enabling objectives mediate the relationship between compounding drivers of conflict and climate stress and the system-level outcomes of equity, resilience, sustainability, and human security. Each pillar is discussed in relation to Syria's post-conflict context in the sections that follow. Together, these elements create the foundation for sustainable oral health systems capable of maintaining essential services under conditions of ecological stress and social disruption (8, 9). Health systems—and oral health services in particular—both depend on these systems and contribute to environmental pressures through energy consumption, water use, materials sourcing, and healthcare waste generation (10). Oral healthcare services should operate within planetary boundaries, embedding environmental sustainability and system resilience into the national oral health strategies and action plans.

In fragile and conflict-affected settings such as Syria, the interactions between planetary health and oral health systems become especially pronounced. Climate change acts as a risk multiplier, intensifying existing vulnerabilities through rising temperatures, water scarcity, increased frequency of extreme weather events, and disruptions to infrastructure and supply chains (11, 12). Oral health services are particularly sensitive to these stressors due to their reliance on patient and workforce mobility, stable electricity for sterilization and refrigeration, access to clean water for infection prevention and control, and uninterrupted availability of consumables and essential materials (6). When these foundational systems are compromised, oral health services are often among the first to be disrupted, amplifying preventable disease burden and social inequities.

Rebuilding oral health systems in post-conflict Syria therefore requires an explicit focus on human security and climate-resilient healthcare systems. Climate resilience in oral health care extends beyond technologically advanced solutions and includes context-appropriate strategies such as energy-efficient facility design, integration of renewable energy sources, water-conserving practices, sustainable procurement, and environmentally responsible healthcare waste management (9). In resource-constrained environments, these measures serve not only environmental objectives but also practical adaptations that enhance service continuity amid chronic infrastructure instability.

Positioning oral health within an oral and planetary health framework necessitates a shift from resource-intensive, restorative-centric models toward protective prevention, integrating oral health promotion and care into primary health care and universal health coverage benefit packages and reducing environmental footprints of oral health care (10, 13).

## 3 Building a sustainable and resilient oral health system

As the country begins the process of recovery and repair post-conflict, Syria's oral health system must be grounded in broader health system recovery strategies that prioritize both human capital, social cohesion and equitable service delivery. Table 1 describes the domains, strategic priorities and system challenges for rebuilding oral health systems in Syria. It aligns with action 8 of the WHO Global Oral Health Action Plan (2023–2030) (6). which calls for the integration of oral health into national emergency preparedness and response frameworks.

**Table 1:** System building blocks for rebuilding oral health systems in post-conflict Syria.

WHO system building block	Strategic priority (WHO global oral health action plan 2023–2030)	Challenges in post-conflict Syria
Service delivery	Integrate oral health into primary care, school health, and emergency services using a tiered approach	Damaged infrastructure, urban-rural disparities, limited service points, lack of integration
	Adopt climate-resilient and environmentally sustainable health care facility standards	
Health workforce	Reform oral health education to include public health and emergency preparedness; expand roles through task-shifting and community training	Displacement of dental professionals, outdated curricula, lack of training in crisis response
	Promote community-based oral health education.	
Health information system	Develop mobile data tools; integrate oral health indicators into national health databases; promote participatory monitoring	Absence of oral health data systems, limited digital infrastructure, weak monitoring capacity
Access to essential medicines	Utilize cost-effective, locally available materials and engage the private sector	Supply chain disruptions, lack of locally sourced materials, limited access to fluoride and hygiene kits
Financing	Advocate for inclusion of oral health in national budgets and donor strategies; engage private sector for cost-effective solutions	Low prioritization in funding, economic instability, limited donor awareness of oral health needs
Leadership/governance	Embed oral health in national health and emergency strategies; define coordination roles across MoH, professional bodies, WHO/humanitarian actors, civil society, and WASH agencies	Fragmented leadership, lack of policy inclusion, weak coordination

The strategic priorities in Table 1. could not only strengthen the robustness and resilience of Syria's oral health system but also contributes to broader health, environmental, and socioeconomic goals (6, 10). New definitions for oral health can catalyze new ways of thinking and working that can provide the conceptual foundation for health system recovery strategies.

Operationalizing this building block requires a defined coordination architecture rather than generic calls for partnership. The Ministry of Health, as the convening national authority, would be responsible for embedding oral health targets within national health recovery and emergency preparedness plans. Professional dental associations and dental schools would hold responsibility for workforce standards, curriculum reform, and continuing education aligned with these targets. WHO country offices and humanitarian health actors would support interim service delivery and technical guidance where state capacity remains constrained. Civil society organizations and community representatives would provide participatory input into service priorities and monitor accessibility and acceptability at the local level, particularly in underserved and geographically fragmented areas. Environmental and WASH (water, sanitation, and hygiene) agencies would coordinate on infrastructure requirements—reliable water and electricity—that oral health service continuity depends on directly. Financing responsibility would be shared between government budget allocation, donor coordination mechanisms, and private-sector engagement for cost-effective materials and equipment, with accountability maintained through routine reporting against the indicators described in the Health Information System building block (Table 1). Structured collaborative governance processes—encompassing sequential stages of joint assessment, initiation, deliberation, and implementation—offer one model for how such multi-actor coordination can move beyond *ad hoc* partnership toward accountable, phased engagement (14). Government commitment, active stakeholder participation, transparent communication, adequate resource support, and shared accountability mechanisms are consistently identified as necessary conditions for such governance arrangements to function (15).

### 3.1 Transdisciplinary and cross-sectoral approaches to oral health and dentistry in Syria

Oral health has been equated with clinical dentistry in Syria, both in public perception and as reflected in the university curriculum (16). The new definitions of oral health by the World Health Organization and FDI World Dental Federation emphasize a broader world view than traditional dentistry and include disease prevention, health promotion, addressing social determinants and services and oral healthcare grounded in safeguarding planetary health (10). Acknowledging this distinction between oral health and dentistry is central to building a resilient and sustainable oral health system in Syria (17). Health and oral health are influenced by a range of factors, including multigenerational trauma experienced by specific cultural, racial, or ethnic groups; lived oppression; and discrimination and poverty. Sustainable oral health recognizes the full spectrum of health and oral health, where oral health is understood to be influenced by a wide range of bio-psychosocial-spiritual perspectives and the healthy functioning and integrity of the Earth's systems (5). Sustainable dentistry can be described as practicing dentistry in ways that are environmentally friendly, socially responsible and accountable, and economically viable within planetary health boundaries and which safeguards the integrity of Earth systems (8). Health is one of the seven dimensions of human security (5). A model for sustainable dentistry can establish a foundation for oral health system recovery in Syria that offers a pathway to strengthen human security, safeguard essential services under ecological stress, and contribute to sustainable recovery in fragile settings facing converging climate and conflict-related challenges (8).

The four pillars introduced in Section 2 (Conceptual Framework) and illustrated in Figure 1—preparedness, prevention, stewardship, and foresight—grounded in WHO Strategic Objectives 1, 2, and 7 affirmed in the Bangkok Declaration (2024) (7), and in the sustainable dentistry model (8), can guide planning and processes which must be shaped by the lessons learned from over a decade of crisis response to ensure that an oral health system is not only functional but capable of withstanding new hazards and threats.

### 3.2 Sustainable oral health: A Syrian perspective

Sustainability in relation to oral health remains an emerging and underexplored area among Syrian dental professionals. The following observations draw on the authors' field experience and professional networks within the Syrian dental community, in the absence of published empirical data on sustainability practices in Syrian oral health care. In oral health service delivery, while solar energy is increasingly recognized as a practical solution in regions with unreliable electricity, broader concepts such as waste reduction, energy-efficient design, and sustainable procurement are not yet widely integrated into dental practice in Syria. This gap is likely due to the limited availability of training resources, the impact of prolonged conflict on infrastructure and supply chains, and the economic constraints that shape clinical decision-making. In oral healthcare, the overwhelming focus is on the disease and condition status domain of oral health with little attention given to the other two domains of physiological status and psycho-social status, as well as moderating factors and driving determinants (18).

However, Syrian dental professionals have demonstrated adaptability and openness to new ways of thinking, innovation, particularly when solutions are cost-effective and locally feasible. This presents an opportunity to introduce principles of sustainable oral health into dental education and practice and could include;

- leveraging locally available materials for dental kits and equipment,
- promoting renewable energy sources such as solar-powered solutions which became very popular in Syria due to the lack of network grid power,
- encouraging reuse and recycling practices to reduce medical waste within a robust waste management system
- A holistic model for oral health promotion directed at infants would support a “village” of caregivers who might include pregnant women and new parents, grandparents, extended relatives, and neighbors, all responsible for the baby's health.

These approaches align with broader goals of environmental stewardship and community resilience. Doing so requires long-lasting partnerships within and outside the health sector, as well as engagement with government, communities, civil society, and the private sector. Evidence from collaborative governance in other resource-constrained settings suggests that such partnerships function best when structured through defined stages of joint assessment, initiation, deliberation, and implementation, rather than left as open-ended engagement (14). Operationalizing sustainable oral health in Syria would similarly benefit from structured co-design and co-production processes for oral health promotion, prevention, and care, adapted to the country's specific institutional and humanitarian context rather than transplanted from unrelated settings.

Oral health workforce education is a key aspect of health systems strengthening (Table 1) as it can improve clinical capacity and redefine the role of health professionals including oral health professionals as agents of public health, emergency response, and sustainable care—important for fragile and post-conflict settings like Syria.

### 3.3 Oral health workforce education: building capacity and a common vision

A resilient and equitable oral health system in post-conflict Syria must be underpinned by a well-trained, adaptable, and equitably distributed workforce that is prepared to function under conditions of crisis and uncertainty. Beyond their clinical role, oral health professionals contribute to human security (5).

The World Health Organization's Global Oral Health Action Plan (2023–2030) underscores the need to reform oral health workforce models to align with public health priorities, universal health coverage, and emergency preparedness (6). In Syria, the prolonged conflict has highlighted both the vulnerability and the latent capacity of the dental workforce. Documented cases illustrate this dynamic: Physicians for Human Rights reported instances of dentists performing procedures far outside their training scope—including maxillofacial surgery—where no other qualified provider was available (19). Consistent with this documented pattern, the authors' own professional experience and networks within the Syrian dental community indicate that such improvised role expansion among dentists and dental students was a recurring, if informal, response to system collapse throughout the conflict. While largely undocumented in the broader literature, these adaptations demonstrate the potential contribution of oral health professionals to crisis response when supported through policy, education, and system integration.

Institutionalizing emergency preparedness, disaster response, and climate resilience within oral health workforce education represents a strategic investment in human security. Integrating competencies related to emergency response, infection prevention in crisis contexts, community engagement, and environmentally sustainable practice enables health workers to maintain essential services during armed conflict, pandemics, and climate-related emergencies (4, 20). Such preparedness strengthens health system absorptive and adaptive capacity, ensuring that oral health services remain functional when they are most needed.

Reforming oral health workforce education in Syria further provides an opportunity to adopt transdisciplinary training models that integrate public health, environmental sustainability, social determinants of health, and humanitarian response principles. Decentralized training, interprofessional collaboration, and community-based practice models enhance system flexibility and reach, particularly in underserved and climate-vulnerable areas (21). Digital learning platforms and international academic partnerships can support workforce development in conflict-affected settings by overcoming geographic and resource constraints, facilitating knowledge exchange, and promoting innovation in sustainable and climate-resilient oral health care. Aligning these educational reforms with national health emergency preparedness strategies ensures that oral health systems contribute meaningfully to broader human security and resilience agendas.

## 4 Discussion

Rebuilding Syria's oral health system is not merely a technical or infrastructural challenge—it is a unique opportunity to reimagine oral health as a pillar of public health, social equity, and system resilience. The devastation caused by over a decade of conflict has exposed the vulnerabilities of treatment-centric models of care. In this context, oral health must be repositioned as a strategic component of national recovery, integrated into broader health, education, and emergency preparedness systems.

Introducing sustainability and climate-resilience requirements into a health system still recovering from acute crisis raises genuine trade-offs. Investment in solar infrastructure, environmentally responsible waste management, or climate-adapted facility design competes directly with more immediate demands for restoring basic service capacity, replenishing depleted workforces, and meeting acute treatment needs. In a resource-constrained environment, sustainability measures are more likely to gain traction where they double as practical adaptations to chronic infrastructure instability—such as solar power addressing unreliable grid electricity—rather than where they are framed primarily as environmental objectives. Governance arrangements proposed in this Perspective also carry implementation risk: fragmented authority, competing humanitarian and governmental actors, and limited institutional capacity may constrain the coordination architecture described in Section 3, even where the roles themselves are clearly defined. Equity implications require particular attention, since urban–rural disparities and uneven humanitarian access mean that recovery investments could inadvertently concentrate in more accessible areas unless deliberately directed toward underserved regions.

This Perspective has several limitations. It is a conceptual and normative argument rather than an empirical study, and does not draw on primary data from Syrian oral health stakeholders. Several claims regarding workforce adaptation and sustainability practices rely on the authors' professional experience and available documentation rather than systematic evidence, as noted throughout. The governance and collaborative-process models referenced were developed in different national and sectoral contexts and are not assumed to transfer directly to Syria's oral health system; they are offered as comparative illustrations rather than validated solutions.

This Perspective's contribution is to reposition oral health as a strategic component of human security and planetary health frameworks in fragile, conflict-affected settings—an area where oral health is typically absent from recovery planning altogether. Future research should build on this conceptual analysis by empirically exploring the perceptions, preparedness, and sustainability practices of Syrian dentists and dental students, generating context-specific evidence to inform climate-resilient and human-security-oriented oral health system reforms.

## Data Availability

The original contributions presented in the study are included in the article/Supplementary Material, further inquiries can be directed to the corresponding author/s.
